# Two Cases Treated by Different Strategies for Common Carotid Artery Dissection with Thrombosis Due to a Type A Aortic Dissection

**DOI:** 10.21470/1678-9741-2019-0336

**Published:** 2020

**Authors:** Wei Ren, Feng Shi, Zhiwei Wang, Jiahui Wang, Jinxing Chang

**Affiliations:** 1 Department of Cardiovascular Surgery, Renmin Hospital of Wuhan University, Wuhan, Hubei, People’s Republic of China.; 2 Department of Radiology, Renmin Hospital of Wuhan University, Wuhan, Hubei, People’s Republic of China.

**Keywords:** Aneurysm, Dissecting, Carotid Arteries, Carotid Artery Diseases, Stents, Thrombosis, Cerebrovascular Circulation, Computed Tomography Angiography

## Abstract

Total arch replacement and stent trunk were performed for two patients. One of these underwent a total bilateral carotid artery replacement in anatomical position while the other underwent partial carotid artery dissection. The first patient demonstrated no neurological complication after surgery and a postoperative computed tomography angiography (CTA) showed bilateral common carotid artery patency. However, the second patient had neurological dysfunction after surgery, while a postoperative CTA showed occlusion of the left common carotid artery. Anatomical replacement for a common carotid artery dissection with thrombus has the potential to significantly improve cerebral perfusion and reduce postoperative neurological complications.

**Table:** 

Abbreviations, acronyms & symbols	
AAD	= Type A aortic dissection
CCA	= Common carotid artery
CTA	= Computed tomography angiography
TEVAR	= Thoracic endovascular aortic repair

## INTRODUCTION

Type A aortic dissection (AAD) may tear the common carotid artery (CCA), which leads to a decreased flow rate in true lumen as well as thrombosis in false lumen, seriously affecting blood supply to the brain. Neurological dysfunction may occur in 6% to 16% of patients living with AAD^[1,2]^. At present, there are various theories about how to treat the CCA dissection caused by type A dissection. In this study, we report two cases of CCA dissection with caval pseudothrombosis treated by different procedures. Both the patients in the present study signed the informed consent and all experimental protocols were conducted according to the Declaration of Helsinki and approved by the Human Research Ethics Committees of Renmin Hospital of Wuhan University.

## CASE REPRESENTATION

### Case 1

**Fig. 1:**
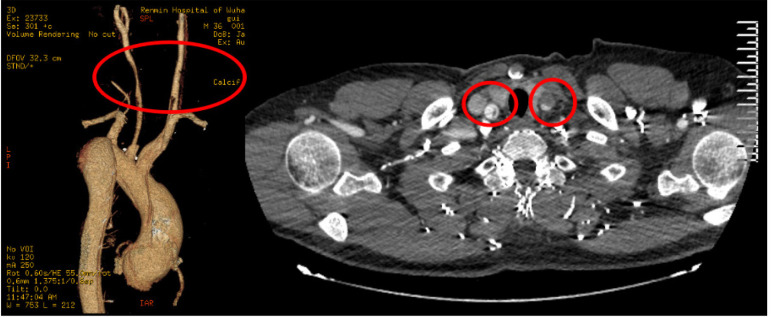
Computed tomography angiography of Case 1 suggesting a type A aortic dissection involving bilateral common carotid arteries (CCAs). The circle indicates a narrow true lumen in the left CCA and a large number of thromboses in the false lumen.

**Fig. 2:**
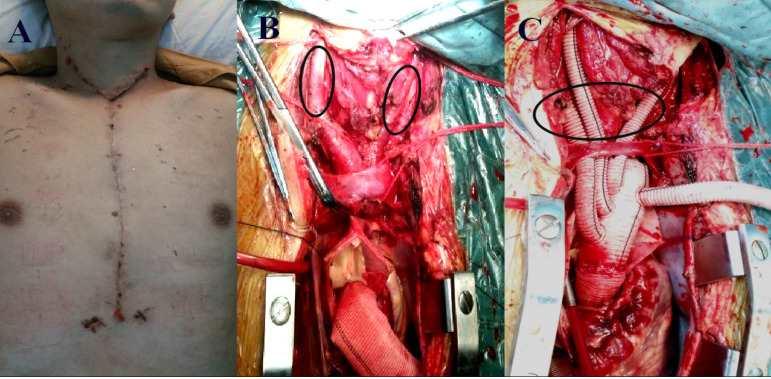
A) Shows the Y-shaped incision in Case 1 to expose the aorta and bilateral common carotid arteries (CCAs). B) Shows the bilateral CCAs being torn by the dissection (indicated by black circle), while C) shows the reconstructed right subclavian artery, as well as the right CCA and left CCA, by vascular prosthesis (from left to right in the black circle). Blood flow to the left subclavian artery was restored utilizing the intraoperative stent fenestration technique, which could not be displayed in the figure.

**Fig. 3:**
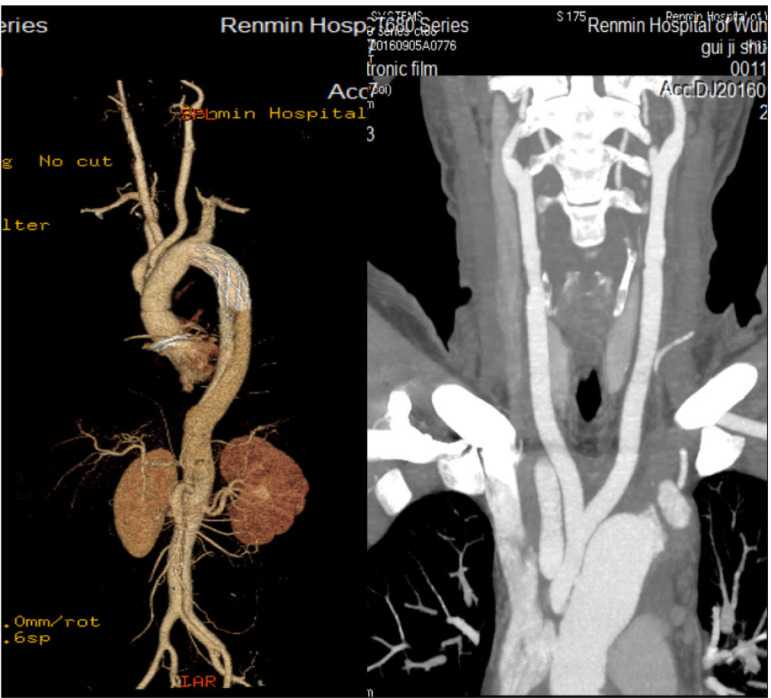
This postoperative computed tomography angiography of Case 1 demonstrates that the artificial vessels of bilateral common carotid arteries had patency without dissection.

A 36-year-old male patient was diagnosed with acute aortic dissecting aneurysm and his chief complaint was severe chest pain for 14 hours, accompanied by transient syncope. He was conscious and without neurological dysfunction when he arrived at the hospital. Computed tomography angiography (CTA) revealed that the dissection was torn from the aortic root to the iliac artery, involving all branches of the arch. In particular, the left CCA was torn into true and false lumens, while a large number of thrombi in the false lumen oppressed the true lumen, resulting in severe stenosis. There was no thrombosis in the false lumen following the tear of the brachiocephalic trunk and right CCA (Figure 1). A computed tomography scan of the head found no obvious cerebral infarction or cerebral hemorrhage. Echocardiography indicated both aortic root aneurysm and severe aortic valve insufficiency. The patient then underwent Bentall surgery plus total arch replacement and stent elephant trunk surgery. A sternotomy was performed in order to expose the branches of the superior arch. The bilateral CCAs were then fully exposed to its bifurcation along the axis of the sternocleidomastoid muscle. Bilateral cerebral perfusion was adopted during deep hypothermic circulatory arrest. A perfusion tube was directly inserted into the brachiocephalic trunk on the right side, while the left tube was inserted in the distal typical part of the left CCA. Four branches of vascular prostheses (Maquet, Cardiovascular LLC, NJ, and US) were used for total arch replacement, bilateral CCA replacement, and right subclavian artery reconstruction. The intraoperative stent fenestration technique was utilized for the left subclavian artery in order to establish blood supply^[3]^ (Figure 2). Ten hours after surgery, the patient recovered completely and had no transient neurological dysfunction and no permanent neurological insufficiency. There was no cervical muscle dysfunction after one year of follow-up. CTA indicated both good blood flow in the four branches above the arch and no dissection in the carotid artery (Figure 3).

### Case 2

**Fig. 4:**
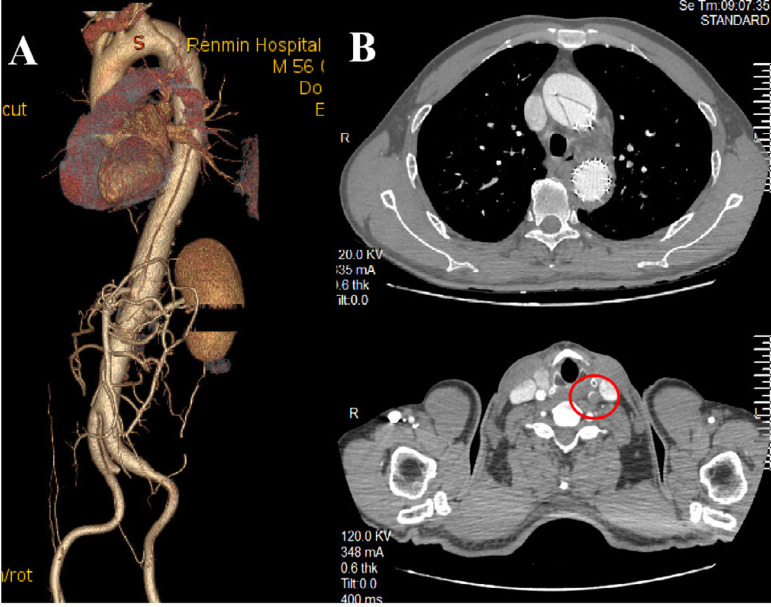
A) Computed tomography angiography of Case 2 showing type B aortic dissection before a thoracic endovascular aortic repair (TEVAR) procedure. B) Shows the retrograde tear after TEVAR forming type A aortic dissection and common carotid artery (CCA) dissection, while the red circle shows a large thrombus in the false lumen of left CCA, leading to a severe narrowing of the true lumen.

**Fig. 5:**
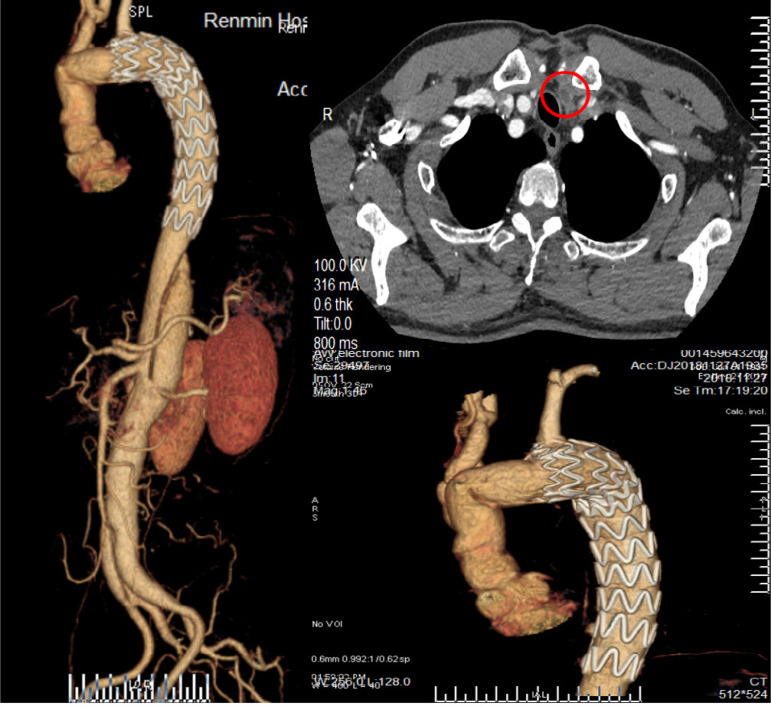
Computed tomography angiography following total arch replacement in Case 2 showed normal development of brachiocephalic trunk and left subclavian artery but no development of the left common carotid artery.

A 56-year-old male patient was admitted to hospital with severe chest and back pain for 10 hours. Initial diagnosis was a type B aortic dissection. Two weeks after admission, the patient still had back pain and CTA indicated that the pseudocavity was large and the true cavity was small, with poor organ perfusion. Therefore, a thoracic endovascular aortic repair (TEVAR) was performed, and the left subclavian artery opening was not closed. On the third day following surgery, the patient complained of pain in the anterior cardiac region with recurrent dizziness. A re-examination of CTA revealed a retrograde tear of the ascending aorta to form a type A dissection. The brachiocephalic trunk was torn by the dissection while the right CCA was normal. The left CCA was torn to the bifurcation and the true cavity was narrow as a result of a massive thrombosis in the distal pseudocavity (Figure 4). An ascending aorta and total arch replacement was then performed under extracorporeal circulation. Due to the large number of thrombi in the pseudocavity of the left CCA, cerebral perfusion was performed with a unilateral brachiocephalic cannula when circulation was stopped. The aortic arch was cut open intraoperatively and a large number of fresh thrombi were found in the left carotid artery pseudolumen, with little blood return. The thrombus was sucked as far as possible into the suction device until there was no visible thrombus and a good blood return. The branch of the vascular prosthesis was anastomosed directly onto the dissecting wall of left CCA. During the early postoperative period, the patient was in a state of severe delirium. However, he recovered completely in approximately 48 hours, without any permanent neurological dysfunction such as hemiplegia. The aortic CTA was reviewed on the ninth postoperative day, which suggested that the left CCA was completely occluded from origin. The brachial and left subclavian arteries developed normally (Figure 5). After 18 months of follow-up, CTA re-examination revealed the same situation. The patient’s main symptom was repeated severe dizziness. Reoperation on the left CCA was recommended but was rejected by the patient.

## DISCUSSION

The frequency of aortic arch branches involved in AAD ranges from 5 to 43%. Gaul et al.^[4]^ have shown that 44 out of 102 patients with AAD have the superior arch vessels involved. Luehr et al.^[5]^ discovered 23 cases of poor cerebral perfusion which had been caused by secondary unilateral or bilateral CCA dissection in 354 consecutive AADs. The management of CCA dissections due to type A dissections remains controversial. In 2013, Charlton-Ouw et al. reported that CCA dissection secondary to aortic dissection had good outcomes following aortic repair surgery, and that no additional surgical treatment was required regardless of the stenosis degree^[6]^. However, some recent studies have suggested that CCA dissection should be treated as soon as possible. In 2015, Luehr et al.^[5]^ found out that for patients with AAD and CCA occlusion, active extra-anatomic revascularization of CCA could better restore cerebral perfusion and reduce the risk of neurological complications. In 2016, Navid published a similar study, demonstrating that CCA dissection associated with aortic dissection plays a key role in stroke and has a poor prognosis. However, a positive CCA replacement strategy can improve neurological prognosis^[7]^.

Cerebral perfusion flow may change in CCA dissection regardless of the cause. Although some patients with AAD have no cerebral perfusion failure before surgery, neurological complications are the focus of prevention for this deep hypothermic circulatory arrest surgery^[8]^. Additionally, it has been reported that following AAD surgery, CCA dissection has developed into complete occlusion, at which time serious neurological complications occur. Even if CCA is again treated with emergency surgery to restore cerebral perfusion, patients’ recovery will be seriously affected^[9]^. When this evidence is viewed in the light of the two cases on which we reported, we believe that CCA replacement should be actively performed for patients with AAD.

There are few studies exploring how to reconstruct such dissecting CCA. Lentini reported on an open/hybrid technique using carotid stents and gelatin-resorcinol-formaldehyde glue binders for the treatment of total carotid dissection^[10]^. However, there is a high cost and a risk of glue entering the true lumen with the break and blocking the intracranial arteries. Luehr et al.^[5]^ used an extra-anatomical bypass to reconstruct CCA through the ascending aorta, while the retained CCA dissection still existed. In this case, bilateral CCA was completely reconstructed anatomically in situ following resection of carotid artery dissection and thrombus, from the beginning of CCA to the normal distal part. Generally, dissection does not involve the internal and external carotid arteries, which are secondary branches of aorta. This means that most typical anastomotic sites do not exceed the bifurcation of CCA. This total carotid artery replacement strategy completely reconstructs blood supply to the brain, including bilateral CCA and bilateral subclavian artery, and it has the ability to directly direct cerebral perfusion to the ischemic side of cerebral vessels during the non-circulation phase. At the same time, the ischemic side of the brain can be directly perfused, meaning that this surgical procedure can easily expose CCA without affecting neck muscle function.

In the second patient, left CCA dissection was not completely replaced in the normal site, resulting in occlusion of left CCA following surgery. The thrombus in the pseudocavity was repeatedly sucked during surgery and the anastomosis was performed when the true cavity was unobstructed and had a good return of blood. However, as the true and false lumens at the distal end of the anastomosis persisted and the collapse of the true lumen was minimal, it is possible that the pressure in false lumen was higher than that in true lumen and that thrombus formed again in the false lumen, leading to the occlusion of CCA. While this patient is fortunate to have no permanent neurological dysfunction following surgery, he still experiences clinical symptoms of cerebral ischemia, which greatly increase the risk of stroke.

## CONCLUSION

Therefore, we recommend that a positive replacement strategy should be adopted for the CCA dissection in AAD. It would be better to completely replace CCA in the case of thrombosis formation in the false lumen of dissection, as this not only ensures accurate anastomosis on the typical vessel wall, but also completely eliminates the false lumen and removes the thrombus in order to improve cerebral perfusion. Of course, this method has only been used in a few cases and has certain limitations. Therefore, further large-scale clinical studies are needed to provide more evidence.

**Table:** 

Authors' roles & responsibilities	
WR	Substantial contributions to the conception or design of the work; final approval of the version to be published
FS	The acquisition, analysis, or interpretation of data for the work; final approval of the version to be published
ZW	Drafting the work or revising it critically for important intellectual content; agreement to be accountable for all aspects of the work in ensuring that questions related to the accuracy or integrity of any part of the work are appropriately investigated and resolved; final approval of the version to be published
JW	Analysis, or interpretation of data for the work; final approval of the version to be published
JC	Interpretation of data for the work; final approval of the version to be published
